# P287: Adherence to biosafety practices by nursing staff in the face of situations of occupational risk

**DOI:** 10.1186/2047-2994-2-S1-P287

**Published:** 2013-06-20

**Authors:** PB Santos, MS Moraes, ACDS Oliveira, S Scotá, AS Gomes, SR Moura, RS Martins

**Affiliations:** 1 Aprimoramento Profissional, Institute of Infectious Diseases Emilio Ribas, São Paulo, Brazil; 2 Diretoria de enfermagem, Institute of Infectious Diseases Emilio Ribas, São Paulo, Brazil; 3 Educação Continuada e CCIH, Institute of Infectious Diseases Emilio Ribas, São Paulo, Brazil

## Introduction

The nursing staff is more susceptible to biological hazards in health services, due to the greater frequency of patient contact. Thus, preventive measures to contagion and spread of disease are essential to provide safe care.

## Objectives

To assess the knowledge and use of Personal Protective Equipment (PPE), as well as the biosafety measures taken by the nursing staff, and the factors interfering in the exercise of such practices.

## Methods

This was an observational, non-experimental, quantitative, cross-sectional, developed at the Institute of Infectious Diseases Emilio Ribas, reference hospital care to infectious diseases. The study population consisted of 52 professionals, including nurses and nursing assistants who worked in one of four adult patient units.

## Results

73,1% (38), followed by isolation aerosols, 65,4% (34), being a significant index of professionals joined the use of respirator mask type, 89.7% (52). Regarding the isolation of contact using gloves and apron simultaneously, showed low adhesion to 10.5% (2) 19 uses observation opportunities. Between the professionals, 25% (13), assumed that do not use PPE at some point in their activities, the main reasons are the discomfort / difficulty breathing, 30.7% (4), and the gloves because they decrease the sensitivity, 30.7% (4). With regard to hand hygiene there was a low uptake in these three conditions: 11.13% (6), before contact with patients, 57.6% (45), after contact, and 52, 3% (11) after removal of gloves.

## Conclusion

Despite having knowledge about the need for the use of PPE in the appropriate caution by contact, professionals had attitudes incompatible with the aforementioned. In the case of isolation by spray gave a significant value for people who know 65.4% (34) and adhere to the mask respirator type 89.7% (52). Regarding hand washing, there was obtained an index unsatisfactory.

## Competing interests

None declared

